# ‘A blender without the lid on’: Mealtime experiences of caregivers with a child with autism spectrum disorder in South Africa

**DOI:** 10.4102/sajcd.v67i1.708

**Published:** 2020-10-29

**Authors:** Skye N. Adams, Raeesa Verachia, Kim Coutts

**Affiliations:** 1 Department of Speech Pathology and Audiology, Faculty of Humanities, University of the Witwatersrand, Johannesburg, South Africa

**Keywords:** autism, family-centred care, feeding, mealtimes, picky eating, South Africa

## Abstract

**Background:**

Evidence suggests that caregivers of children with autism spectrum disorder (ASD) and associated feeding difficulties have specific mealtime challenges in the home environment because of the limited interventions that are appropriate and responsive to the needs of the child as well as the family.

**Objectives:**

To describe: (1) common feeding difficulties in children with ASD, (2) mealtime challenges for the caregiver and (3) mealtime strategies used by the caregiver.

**Method:**

Forty caregivers were purposively sampled to participate in the study. Participants were recruited through ASD-specific schools in Johannesburg, South Africa. Participants completed an online questionnaire focusing on their mealtime experiences with their child with ASD.

**Results:**

Findings from the data after thematic analysis indicated the multitude of challenges caregivers have when feeding their child with ASD as well as their individualised way of dealing with these difficulties.

**Conclusion:**

The findings emphasised the importance of incorporating the family’s beliefs, values and needs into feeding management and highlight the importance of a holistic approach to intervention. The study also provided information about mealtime strategies that are being used in the home environment to support the child as well as the caregiver. This information can be used to inform management to improve therapeutic outcomes and feeding challenges in the home environment.

## Introduction

The prevalence of autism spectrum disorder (ASD) is increasing globally, requiring improved service delivery (Eslabbagh et al., 2012). The exact prevalence of ASD in South Africa is unknown; however, Lamb (2018) has estimated that currently over 1 million individuals are living with ASD in South Africa. Children with ASD present with a wide range of associated difficulties, including communication, behaviour and those related to feeding. Research has shown that 46–89% of children with ASD will present with feeding difficulties (Ledford & Gast, 2006), which may be related to problems with food acceptance, food selectivity, food progressions and challenging mealtime behaviours (Bandini et al., 2010; Gray et al., 2018; Matson & Fodstad, 2008; Nadon, Feldman, Dunn, & Gisel, 2011). Children are at risk of a number of nutritional and developmental inadequacies if feeding difficulties are not managed or resolved. Children with ASD may also experience contextually specific feeding difficulties that may differ at school, in therapy and at home. Thus, there is a need to improve understanding of the feeding challenges associated with ASD in a range of contexts to better inform feeding interventions.

### Family mealtime routines and rituals

Mealtimes are a complex process that constitutes both the cultural and social commensality of family routine. Commensality is the practice of eating together and sharing food (Ochs & Shohet, 2006; Tuomainen, 2014). The most fundamental of commensalities are those related to family mealtimes. In many societies and communities, mealtimes are seen as a symbol of unity and family stability (Hamilton & Hamilton Wilson, 2009). Each household will have their own way of expressing mealtime routine, rituals and patterns. In majority of families, the caregivers have a strong influence on their child’s eating behaviours (Quansah, Ohene, Norman, Mireku, & Karikari, 2016; Scaglioni, Arrizza, Vecchi, & Tedeschi, 2011). Children with ASD may present with feeding difficulties that in turn may influence the family’s mealtime process because of their specific dietary requirements and feeding needs. If the feeding needs of the children with ASD are not met, then the child may refuse to eat (Ausderau & Jaurez, 2013; Bagby, Dickie, & Baranek, 2012). This raises pertinent questions as to whether children can influence family mealtimes and to what extent this impacts the caregiver and the mealtime process. Thus, it is important to explore this phenomenon in the context of the home environment, as this will allow for a greater understanding of the impact of context and culture on feeding difficulties in children with ASD as well as the caregiver.

### Impact of feeding on the caregiver

Children with ASD present with a number of associated difficulties that can negatively influence the family life and functioning within daily routines. Studies have indicated that caring for and feeding a child with ASD place added stressors on the family because of the multitude of difficulties that children may exhibit (Ledford & Gast, 2006; Nadon et al., 2011; Schreck & Williams, 2006). Research that has explored this phenomenon have highlighted that many caregivers will feel an increase in stress, anxiety, stigma and depression when caring for children with ASD (Ausderau & Juarez, 2013; Blanche, Diaz, Barretto, & Cermak, 2015; Gray, 2002; Hutton & Caron, 2005; Suarez, Atchison, & Lagerwey, 2014; Van Tongerloo, Van Wijngaarden, Van der Gaag, & Lagro-Janssen, 2015; Rogers, et al., 2012; Shu, 2009). Thus, it may be understood that caregivers’ raising a child with ASD may experience greater caregiver burden and may be more susceptible to depression and anxiety disorders (Lai, Goh, Oei, & Sung, 2015). However, there is a paucity of local and international research on the impact the mealtimes may have on caregivers, specific caregiver mealtime experiences and the strategies that may be used to assist them. In addition, research has failed to look at the experiences of caregivers from more diverse contexts in low and middle income countries (LMICs) like South Africa. Caregivers living in South Africa may have their own cultural structuring of the mealtime with regard to certain customs and rules. Thus, understanding the experiences of the caregivers who are living in diverse contexts is vital when trying to obtain a holistic view of caregiver experiences when feeding a child with ASD.

### Mealtime strategies

Research has indicated that context and culture will influence intervention compliance and outcomes (Hwang & Charnley, 2010). Literature has also shown that feeding management provided by the therapist will be unsuccessful if it is not sensitive to the needs of the family and does not incorporate the families’ own cultures, beliefs and values (Ausderau, John, Kwaterski, Nieuwenhuis, & Bradley, 2019; Ochs & Shohet, 2006). To date, there is limited research into feeding interventions that are appropriate within the context of the home environment in South Africa. When working with children, interventions require a family-centred approach to care, taking into account not only the needs of the child but also those of the family. In particular, from the key areas outlined above, the following three objectives were formulated:

- To describe common feeding difficulties in children with ASD
- To describe mealtime challenges for the caregiver
- To describe mealtime strategies used by the caregiver.

## Method

### Study design

An exploratory qualitative study design was employed using a questionnaire to meet the aims of the study. Participants were purposively sampled from different ASD-specific schools in South Africa. Recruitment of participants was done using a questionnaire that was available both as an online form and as a printed hard copy to increase the participant response rate (Appendix 1). Data were collected from the caregivers who met the following inclusion criteria: (1) having a child with a primary diagnosis of ASD, (2) having a child between 3 and 10 years of age and (3) reported some form of feeding difficulty from the caregiver. This age range was chosen because by the time the child is 3 years of age, they should be accepting a wide variety of food types and textures, and using a caregiver-reported questionnaire is still appropriate for children up to the age of 10 years (Seiverling, Towle, Hendy, & Pantelides, 2018).

### Participants

A total of 164 questionnaires and links were sent out to caregivers with a total of 40 responses received (24% response rate). Participant descriptions and characteristics are described in Table 1. Thirteen participants (32.5%) filled in a hard copy of the questionnaire and the rest submitted online. Caregivers who participated in the study were between 18 and 65 years of age with the average age range between 30 and 35 years (33.3%, *n* = 13). Those who answered were predominantly mothers (84.6%, *n* = 34). In addition, 70% (*n* = 28) of the caregivers were formally employed and working in a variety of full-time positions, with the remainder being unemployed. This highlights the economic diversity of the participants, which is pertinent for the results of the study.

**TABLE 1:** Participant description and characteristics.

Characteristics	*N*	%
**Parent**		
Age (years)	18–65	-
**Province**		
Gauteng	33	82.5
Mpumalanga	2	5
KwaZulu-Natal	3	7.5
Eastern Cape	2	5
**Employment**		
Employed	28	70
Unemployed	12	30
**Relationship with child**		
Mother	33	82.5
Father	2	5
Grandmother	1	2.5
Brother	1	2.5
Educator	1	2.5
Aunt	1	2.5
**Child**		
Age (years)†,‡	3.0–9.6	-
**Gender**		
Male	33	82.5
Female	7	17.5
**ASD severity**		
Level 1	9	22.5
Level 2	18	45
Level 3	13	32.5

ASD, autism spectrum disorder; M, mean; SD, standard deviation.

† , M = 7.2;

‡ , SD = 1.2.

Note: Level 1, requiring support; Level 2, requiring substantial support and Level 3, requiring major support.

All the families in the study reported only one child with ASD between the ages of 3.0 and 9.6 years with a mean age of 7.2 years (standard deviation [SD] = 1.2). Most of the children were male. With regards to ASD severity, 22.5% (*n* = 9) presented with level 1, 45% (*n* = 18) with level 2 and 32.5% (*n* = 13) with level 3 (Table 2). Autism spectrum disorder severity and functioning levels were determined using the Diagnostic and Statistical Manual of Mental Disorders, 5th Edition (DSM-5) criteria and associated severity ranking reported by the caregivers (American Psychiatric Association, 2013; Matson & Fodstad, 2008). According to the DSM-V criteria, children are characterised according to three levels of severity: Level 1, requiring support; Level 2, requiring substantial support and Level 3, requiring major support (Esler & Ruble, 2015). The severity can be used as a predictor in terms of the associated problems the child may present with; however, it is important to note that this is not an indicator on the impact the diagnosis may have on the caregiver or family.

**TABLE 2:** Themes and sub-themes.

Theme	Sub-themes
(1) Common feeding challenges in children with ASD	(1) Picky eating (2) Eating times (too fast and too slow)
(2) Caregiver mealtime challenges	(1) Eating together (2) Financial implications (3) Unsupportive friends and family
(3) Mealtime strategies	(1) Negotiating (2) Positive reinforcement (3) Television (4) Ignoring the child’s behaviour

ASD, autism spectrum disorder.

### Procedures and data collection

All participants were provided with consent forms before completing the questionnaire. Written and online consents were obtained. There was no risk of harm to the participants, who were also informed of their right to withdraw at any point in the study.

Data were collected from six ASD-specific schools; one private school (Gauteng) and five government schools (Gauteng [*n* = 2], Mpumalanga [*n* = 1], KawZaulu-Natal [*n* = 1] and the Eastern Cape [*n* = 1]). in South Africa. The schools are all for children with a confirmed diagnosis of ASD. The questionnaire consisted of demographic information on the caregiver and child, as well as six open-ended questions. The use of a questionnaire was deemed appropriate as it was cost-effective, convenient and allowed the inclusion of participants from different provinces in South Africa. The questionnaire allowed participants to respond in their own time, and there are no limitations to geographical location of where the participants live. The original questionnaire was piloted and reviewed for validity. Four experts who had experience in working with children with ASD reviewed the questionnaire. After review, all experts commented on the relevancy and appropriacy of the questionnaire, and no changes were made. This improved the face validity of the questionnaire.

### Data analysis

Inductive thematic analysis was used as suggested by Braun and Clarke (2013). The researchers also engaged in reflective note-taking throughout the research process to acknowledge and record any bias. All the responses were recorded verbatim and then coded inductively on a line-to-line basis. Ideas that were recurring in a large number of the transcripts or those that related to the research questions were categorised. These categories were then grouped into themes and sub-themes. The second author then validated the themes and analysed all the transcripts independently to ensure inter-coder agreement. Inter-coder agreement was established when the same categories were reached by the second author (Braun & Clarke, 2013). All the themes and sub-themes were then discussed and agreed upon by all three authors.

### Trustworthiness

To ensure ethical and trustworthy methods, the following steps outlined by Schwandt, Lincoln and Guba (2001) were considered. Dependability was determined by detailing all aspects of the methodology and data analysis to replicate the study. Investigator triangulation was also used by including the perspectives of all three authors in the data analysis. Confirmability was established through reflexivity to reduce the risk of bias. Researcher reflexivity was important, and each researcher independently reflected on their own positionality within the social context being studied. During the data analysis, all researchers’ viewpoints were discussed and consensus was then made on the final themes.

### Ethical consideration

This study was approved by the University of Witwatersrand, Department of Speech Pathology and Audiology internal Research Ethics Committee (HREC Non-Medical) (STA_2019_01) and the Department of Education. All participants provided written informed consent.

## Results and discussion

The results will be described according to the study aims. The following themes were identified as seen in Table 2.

### Theme 1: Feeding challenges in children with autism spectrum disorder

Many children in the current study presented with associated feeding challenges and struggled with family mealtimes with regard to foods eaten and feeding times. Thirty-five per cent of caregivers reported no mealtime challenges whilst the remaining 65% reported some form of difficulty experienced. Caregivers who reported difficulty had children who were in the younger as well as older age range. Although many children with ASD and associated difficulties will improve as they become older, there are a number of children who do not manage or resolve their feeding challenges and end up with persistent feeding challenges. In addition, children in the current study who were grouped as less severely affected by ASD (Levels I and II) presented with less associated feeding difficulties. Some studies have reported that there may be a strong predictive relationship with an increase in children’s repetitive and ritualistic behaviours and parent-reported difficulties (Johnson et al., 2014; Sharp et al., 2013). However, there is limited literature to support the finding that there is a correlation between ASD severity and feeding difficulties in children with ASD. Those children who experienced feeding challenges will be discussed in greater detail in the following section.

#### Picky eating

Amongst the caregivers who reported feeding challenges, 73% stated that they struggled with feeding their child because of their child’s picky eating and resultant restricted diet. Caregivers commented that their children preferred to eat only certain types of food related to taste, texture and/or colour:

‘The only part that concerns me is that he is so choosy about what he eats. He lives off cereal, noodles, yoghurt and loves pastas. If he does not like something he won’t eat it. He mainly eats love burgers, pizza and pasta. So I give him that.’ (Participant 7, Mother, Gauteng)

‘He is fixed into eating savoury food that is green, white and brown. And would eat colour only on fruits. So I stick to that. I don’t know what else to do.’ (Participant 38, Mother, Gauteng)

Further, caregivers reported that they had to ensure that favoured foods were available for their child, even if this meant that different meals had to pre-prepared:

‘We just have to make sure there is always cereal and milk at home as well as noodles. If he doesn’t eat or won’t eat the food (I make) he will eat cereal or noodles.’ (Participant 2, Mother, Gauteng)

‘To ease stress and frustration I prepare food that I know he will enjoy and have separate food for me and my husband and my other boy.’ (Participant 27, Father, Gauteng)

These findings support the notion that many caregivers are struggling with their child’s restricted food selections and are unsure how to provide a more nutritionally balanced diet (Nadon et al., 2011; Seiverling et al., 2018). Caregivers find their child’s picky eating to be frustrating, and many find it easier to give the child food that they will eat, even though these may not be what the rest of the family is eating. Furthermore, these findings highlight how children with ASD can directly impact the foods that families buy and prepare with regard to the extra planning of mealtimes and cost associated with food items.

#### Eating times (too slow or too fast)

Participants in the current study reported feeding challenges related to eating times with regard to both eating too slowly and eating too fast. In all, 43% of caregivers raised eating times as a cause of concern. The reason for children eating slowly could be attributed to a number of reasons such as oral sensitivity, reduced oral motor control and/or behavioural factors (Nadon, et al., 2011; Sieverling, et al., 2018). Many caregivers felt annoyed at the increased time it took to complete mealtimes (longer than 30 min):

‘I feel good when I see my son eating nicely enjoying his meal but sometimes especially mornings I feel very annoyed when he eats very slowly when it’s time to go to school.’ (Participant 14, Mother, Gauteng)

An interesting finding in the current study was the concerns of caregivers about their children who ate too quickly. This was related to concerns that their children were not chewing their food properly and may choke or vomit. One mother commented that her child’s quick eating created a lot of mess and described it as being *a blender without the lid on*. Another caregiver stated:

‘My child eats very fast, wish that she could eat slowly to allow to chew… In public places like restaurants she will eat so fast with excitement our fears are that she may choke or vomit.’ (Participant 14, Mother, Gauteng)

The findings support the literature regarding children with ASD taking longer to eat, not self-feeding and not eating enough food (Malhi, Venkatesh, Bharti, & Singhi, 2017). However, the current study also reported on children eating too fast and making a mess; these findings have not been explored in much detail in previous literature. In the study, children who ate too quickly were also classified as being independent eaters, and caregivers were concerned that if they assist their child to eat and decrease the speed that they would then remove their child’s independence. It is therefore imperative that caregivers understand not only the feeding challenges but also the impact that it can have on the child if not managed appropriately; more input is required on different intervention strategies for this type of eating challenge. Furthermore, it is important to acknowledge what is more important for the caregiver and how all of these can be incorporated into intervention. This finding perhaps also emphasises the need to have multidisciplinary team input for intervention for feeding challenges in children with ASD.

### Theme 2: Caregiver mealtime challenges

Caregivers who reported no difficulty generally stated that their child was *an independent eater* or *loved all food* and did not have any challenges. Interestingly, caregivers also stated that even though their children still had a restricted diet that if they were able to sit at the table or eat without making a mess, then they did not consider their feeding habits to be a challenge. Caregivers who did report on feeding difficulties described mealtimes as being *difficult, stressful, very messy* and *challenging* and one that many caregivers dreaded. This is further explored in the following sections.

#### Eating together

One of the most significant findings in the present study was the caregiver’s perceptions of what a successful mealtime was. Caregivers reported that to them a successful mealtime was when all family members sat at a table, ate together and shared their stories. Many caregivers reported that the child with ASD would refuse to sit at the table or they would disrupt the rest of the family during mealtimes with a preference for eating alone:

‘It is stressful. I would love him to have a nice hearty meal with his sister and myself and dad. He refuses to try “normal” food and his reaction really makes me sad. Mealtimes are divided because sometimes even the sight of our food will upset him. We would love to sit at the table and all eat together.’ (Participant 32, Mother, Eastern Cape)

The findings of this study are corroborated by other studies that have emphasised the importance of family mealtimes and using them not only as a way to eat but also to socialise and connect with one another (Ausderau & Juarez, 2013; Ochs & Shohet, 2006). In all, 86% of the caregivers in the current study felt that eating at the table with the rest of the family was the most pertinent issue, regardless of other feeding challenges such as preference for certain foods or additional preparation time. This was echoed in a study by Suarez et al. (2014) where many mothers felt that they were missing a family connection and opportunities to connect as a family when their child with ASD did not eat with the rest of the family. Therefore, there is a need to transform mealtimes to suit the family needs and also take into account the needs of the child. Intervention needs to acknowledge that intervention needs to be multifaceted and take into account the needs of the child and the family.

#### Financial implications

This was a significant finding as 30% of the caregivers in this study were unemployed. That said, financial implications for feeding was a significant finding for 70% of caregivers who struggled to feed their child-specific foods and accommodate the food needs of the rest of the family. This implies that even those caregivers who had an income experienced financial concerns in terms of dietary management. The child’s restricted diet often resulted in caregivers having to cook two meals, which resulted in financial stress and burden for the caregiver. However, there were also a few caregivers who were unable to provide the child with their preferred foods and had to force the child then to eat what the rest of the family was eating:

‘It is difficult because of money and that food is not good but that is what he wants. It is so much money.’ (Participant 13, Mother, Gauteng)

‘It is really a challenge because he likes certain things and sometimes I don’t have the money to buy his favourite food, so I have to force him to eat.’ (Participant 22, Mother, Gauteng)

The findings of the financial burden of fussy eating support the impact that context and culture can have on family mealtimes. In South Africa, there are a number of families living with food insecurity and has been largely ignored by researchers in the field (Guler, De Vries, Seris, Shabalala, & Franz, 2018; Rogan, 2016). Many caregivers living in South Africa have financial difficulties, and children with feeding challenges can increase the financial strains because of the caregiver spending money on food as well as food waste. More support and education are required to assist families with possible food insecurity challenges, especially in a context like South Africa. Therapists also need to be more cognizant of these challenges when recommending intervention strategies.

#### Unsupportive Friends and Family

In this study, 40% of caregivers felt a lack of support from their family. This made feeding difficulties in their child more pronounced and limited with regard to where they can go to eat and who they can visit. This further restricted mealtimes to the home for families of children with ASD:

‘Some family members do not understand him and they are impatient with him. This hurts me.’ (Participant 24, Mother, Gauteng)

‘As a mom I have to adjust and go with his flow because no one will understand him better than me in the family but negative impact is that my parents are not with me always to give me support with my boy as I am facing lot of challenges with our neighbours and even some of my friends.’ (Participant 14, Mother, Gauteng)

Because of the lack of support from family and friends, many caregivers reported that they chose to avoid certain places or events because of the challenges experienced with their child’s feeding and eating behaviours. This avoidance can result in further exclusion and isolation for the caregivers and the child. These findings highlight the importance of a strong support system and the impact that the absence of one can have on the emotional, social and psychological well-being of caregivers. In addition, if caregivers are experiencing any negative feelings, this can further result in reduced support for their child. A caregiver needs to be able to take care of themselves before they can adequately take care of their child (Hastings et al., 2005). Support for the caregiver should be incorporated into the management plan by all team members, when working with children with disabilities.

### Theme 3: Mealtime strategies

In this study, 70% of caregivers reported that their mealtimes required some sort of adaptation in their typical family routine to promote eating and mealtime participation. Strategies were often aimed at trying to encourage a mealtime that mirrored a more ‘typical’ representation of what caregivers thought mealtimes should be. Thus, strategies focused on having the child sit with the rest of the family, eat what the rest of the family is eating and reducing mealtime distractions promote a more socially inclusive environment. Caregivers also used strategies to try and alleviate their own stressors (Table 3).

**TABLE 3:** Mealtime strategies used by caregivers to manage feeding difficulties in children with autism spectrum disorder.

Strategy	Definition	Supporting quote
Negotiating	Caregivers responded to a child’s difficult feeding behaviour by negotiating or offering the child a preferred toy or food item	‘I make a deal with him that after he finished with eating I will go buy McDonalds for him or tells him that his father is coming so he will drive with him anywhere he wants to go.’ (Participant 6, Father, Gauteng)
		‘My son is obsessed with sunglasses. If I give him them or either roll-on deodorant he can feed himself nicely and finish every part his meal.’ (Participant 14, Mother, Gauteng)
Positive reinforcement	Hugs and kisses to promote constructive and reinforce behaviour that caregivers deemed to be positive such as trying to make the child to co-operate in a way that facilitated more participation from the child during mealtimes or to achieve desired behaviours and feeding outcomes	‘I give him hugs and kisses to get him to eat all of his food. I also kiss him to show him he did a good job of eating everything.’ (Participant 17, Mother, Gauteng)
Television	Television was used as both a distraction and a reward to try and make their child to finish their meal or stay seated whilst eating	‘We will eat whilst watching TV. TV causes him not to focus on the food but to eat whilst watching TV.’ (Participant 1, Brother, Gauteng)
		‘She loves TV If I can see that she does not want to eat I switch off the TV and feed her. I can tell you that she will finish without any problems.’ (Participant 4, Mother, Gauteng)
Ignoring the behaviour	Response of caregivers to problematic mealtime behaviours such as crying, screaming and throwing tantrums. Caregivers reported that they would ignore and not respond to their child’s behaviour until it is stopped	‘It can be little difficult as we don’t always know how to best handle situations where he is screaming or shouting. Is he is eating and running around we will ignore him until he comes and sits down again.’ (Participant 37, Mother, Kwa-Zulu Natal)

Caregivers struggled to include their child with ASD in the family mealtime, and this caused great distress as the focus and representation for family mealtimes were to sit together and share a meal. Previous research on feeding management has always focused on improving restrictive eating and the child with ASDs acceptance of non-preferred food items (Koegel et al., 2012; Seiverling, Williams, Sturmey, & Hart, 2012; Stough, Gillette, Roberts, Jorgensen, & Patton, 2015). However, the current study highlighted how caregiver’s strategies were aimed at not only getting the child to increase their food selectivity but also to stay at the table, engage with family members or eat a greater quantity of a preferred food. One caregiver commented that her child had very selective eating but reported no mealtime challenges, as he was able to sit at the table with the rest of the family. When managing feeding difficulties, Ausderau et al. (2019) explored the strategies parents use to support the mealtime participation of their child with ASD and found similar findings in mealtime strategies being used at home such as using parent-directed strategies. Further, both the study by Ausderau et al. (2019) and the current study emphasise that although the intake of new foods is important to many parents, and often the focus of many speech language therapy feeding interventions, it is not always the caregivers’ main area of concern or focus.

Therefore, the findings emphasise that therapists should be aware of feeding difficulties in children with ASD reported in the current study. Therapists should also be aware of the impact of mealtimes on the caregiver and the associated challenges some caregivers may have. Further, therapists should incorporate the investigation of current feeding strategies being used by the caregivers to use them as a starting point to provide further support and assistance.

## Limitations and future directions

The authors are aware of a number of limitations of the current study. Firstly, there was no quantitative measure of stress and quality of life for the caregivers in this study; in addition, using a self-administered form did not allow for the researcher to probe on certain topics. However, the researchers wanted to access participants from different geographical locations and use a method that was both convenient and cost-effective; thus the use of a questionnaire was deemed appropriate. Secondly, the authors are also aware of the language limitations and that caregivers were required to be proficient and literate in English. English is not the main language of many South African families. Lastly, all children in the current study were attending school. There may be differences to children who are not in school and the subsequent feeding difficulties experienced by their caregivers. Although, schools were chosen as children all have a confirmed diagnosis of ASD. Future research should include more interviews and observational data to obtain a more holistic picture of the challenges as well as the way in which strategies are being used by the caregivers when feeding their child with ASD. The current study also focused on the caregiver report, primarily mothers. Including additional family members could enhance the understanding of mealtime experiences with a child with ASD in the South African context.

### Implications

The following recommendations are drawn from the research and situated within a family-centred approach:

- Therapists need to be aware about the associated feeding challenges in children with ASD, which may have benefits that are twofold: firstly, for early identification and treatment and, secondly, the opportunity for more support and education for families of children with ASD.
- The experiences reveal that their needs are not always considered when targeting feeding in children with ASD and the need for more contextually relevant and responsive interventions as well as the incorporation of each families’ individual feeding routines, beliefs and values into intervention

## Conclusion

This study has shed some light on the experiences of caregivers feeding their children with ASD within the South African context. A noticeable strength of the current study is that the sample population and the focus on caregivers from LMICs such as South Africa are novel and necessary to transform therapist management. These findings highlight the multitude of challenges that caregivers have when feeding their child with ASD and the different mealtime strategies that they use. These findings have assisted to identify the gaps in the field, which require further exploration.

## Appendix 1:

### List of questions:

1. What feeding difficulties does your child with ASD present with? Please describe how these impact on mealtimes with you and your family?
2. As a caregiver of a child with ASD, how do you feel about the feeding process and mealtimes?
3. How does it impact on you and your family?
4. Are there any strategies or methods that you use to manage your child’s feeding difficulties? Please elaborate.
5. Do the feeding difficulties restrict you from going to certain places or participating in particular activities? If yes, what?
